# Supplementary material for: Internal and External Validity of Social Media and Mobile Technology-Driven HPV Vaccination Interventions: Systematic Review Using the Reach, Effectiveness, Adoption, Implementation, Maintenance (RE-AIM) Framework
Source: Vaccines (Basel). 2021 Feb 26;9(3):197. doi: 10.3390/vaccines9030197 (PMC7996801; doi:10.3390/vaccines9030197)
Supplement: Supplementary file 1 [file vaccines-09-00197-s001.pdf]

vaccines

MDPI

Supplementary Table 1: RE-AIM Dimensions and selected indicators reported by the reviewed articles (n =17)

Author/year/ Country	Design/ Outcome	Reach	Efficacy	Adoption	Implementation	Maintenance
**Chodick et al** (2020) **Israel**	• Design: RCT<br>• Outcome: HPV vaccine uptake	• Target Population: Mothers with 14-year-old daughters<br>• Behavior: Not described<br>• Sample (n = 21,592)<br>• Recruitment: No specific recruitment method was described but mothers who were members of Maccabi Healthcare Services were recruited to the study.	• Uptake: 55.3% vaccine uptake in Intervention group vs 55.0% in control.<br>• Increased in vaccine uptake in Higher SES Facebook campaign group 55.8%<br>• Condition: Facebook campaign group (n=17,271) vs control condition (n=4,321)<br>• Assessment: Post assessment of outcome<br>• Vaccinated = 9551+2377=11928	• Setting: Hospital setting (Maccabi Healthcare Services)<br>• Staff: Gynecologist developed the campaign messages	• Medium: Facebook was used to deliver the content and videos<br>• Theory: Inoculation theory<br>• Duration: No specific time frame but participants review the content on Facebook and watch the video<br>• Cost: Total cost of intervention not reported	• Institutionalization: No Data
**Fontenot et al** (2020) USA	• Design: Pilot Intervention<br>• Outcome: HPV vaccine uptake	• Target Population: MSM 18-26 years<br>• Behavior: unvaccinated, not up to date, no vaccine status.<br>• Sample (n = 42)<br>• Participation rate:42/54= 78%<br>• Recruitment: Mobile app recruitment through MSM dating app.	• Uptake: 23% vaccine uptake<br>• Condition: Single group pre/post-intervention<br>• Assessment: Pre/post assessment of outcome<br>• Vaccinated =10	• Setting: Communities in Boston, Massachusetts<br>• Staff: Intervention delivery staff was not described but hospital staff involved in the data collection process	• Medium: mHealth tool<br>• Theory: Implementation intention theory and design thinking<br>• Duration: No specific time frame but participants review the mHealth tools at their own pace<br>• Cost: Total cost of intervention not reported but participants received $5 gift cards	• Institutionalization: No Data

					plus a chance to win $75 gift card	
**Gerend & Madkins et al**. (2020) USA	• Design: RCT<br>• Outcome: HPV vaccine uptake	• Target Population: Young Sexual Minority Men 18 -25<br>• Behavior: Unvaccinated<br>• Sample (n = 150)<br>• Participation rate: (150/155) 96.77%<br>• Recruitment: Recruited participants using social media and advertisement	• Uptake: Vaccination initiation higher among the intervention group (19.4%) vs. control group (6.6%)<br>• Condition: RCT: Intervention group (n=72) vs. control group (n=76)<br>• Assessment: Assessed vaccination status at baseline, 3-week follow-up and 9-month follow-up assessments.<br>• Vaccinated =14+5 = 19	• Setting: No specific setting but participants were recruited from Chicago areas.<br>• Staff: No intervention delivery staff description	• Medium: Used text messages to deliver intervention messages.<br>• Theory: Information-Motivation-Behavioral Skills (IMB) model framework<br>• Duration: Intervention duration or contact was daily for the first 3 weeks and changed to 1 per month for 8 months.<br>• Cost: Total cost not reported but each participant could receive $75	• Institutionalization: No Data<br>• However, 9-month follow-up was conducted<br>• Attrition rate: between 4% -7% attrition rate at 3 weeks follow up and 9% -12% at 9 months follow-up
**Gerend et.al** (2020) USA	• Design: Not stated<br>• Outcome: Increase in overall HPV vaccination rates	• Target Population: Male and female students<br>• Sample (n = 799)<br>• Behavior: had not yet completed the HPV vaccine series<br>• Recruitment: No specific recruitment strategy described.	• Observed a 75% increase in HPV doses.<br>• Observed a trend that more HPV vaccine doses were administered to students older than 26 years of age in 2019 vs 2018<br>• Intervention condition was not described but it was a single group intervention<br>• Baseline assessment and post intervention assessment	• Setting: University campus<br>• Staff: UHS staff consisted of physicians, physician assistants, and nurse practitioners delivered the intervention	• Medium: Weekly social media postings (Not mention specific social media)<br>• Theory: No Theory<br>• Duration: Multi-intervention components which had 30-minute PowerPoint presentation<br>• Intervention has 2 components: (1) student direction	Institutionalization: No Data

Note: RCT = Randomized Controlled Trial; MSM = Men having sex with men;

			• Vaccinated =599		campaign materials (2) provider directed training and HPV vaccination encouragement<br>• Intervention was limited to the first three months of the Spring semester<br>• Cost: No data	
**Kempe et al.** (2016) USA	• Design: RCT<br>• Outcome: Increase HPV vaccine completion series	• Target Population: Parents of eligible adolescents receiving their first HPV vaccine<br>• Adolescents (males and females) ages 11-17; PCO members for past 2 years Sample (n = 929)<br>• Behavior: Already received 1st dose<br>• Recruitment: Active enrollment with intervention group, passive enrollment in control group	• Uptake: Significant increased vaccination completion rate among intervention group compared to control group (63% vs 38% respectively)<br>• Intent-to-treat analysis<br>• Condition: RCT: cluster, randomized pragmatic trial (intervention n=374 or control group n=555)<br>• Assessments: Two follow-up assessments<br>• Vaccinated = 236+211=447	• Setting: KPCO Clinic<br>• Staff: Clinic staff including pediatric, nurses, medical assistants who helped in enrollment phase.	• Medium: Used text messages, email, or auto-dial:<br>• Theory: No theory<br>• Duration: Not specific but KPCO used an Interactive Voice Response (IVR) system, which is capable of producing multiple automated recall messages parents selected reminder recall method<br>• Recalls issued for each remaining dose<br>• Cost: No data	• Institutionalization was part of the long-term plan of the researchers.<br>• Series completion rates were measured 1 year after HPV dose 1 was received
**Kim et.al** (2020) USA	• Design: RCT<br>• Outcome: measure increase/change in initiation of	• Target population: Korean undergraduate and graduate female students living in the USA<br>• Behavior: Had not yet received HPV vaccination	• Vaccine uptake: Intervention group was twice as likely to receive HPV vaccine dose compared to control group.<br>• Other impact: Both condition increased knowledge.	• Setting: Colleges, churches, social media.<br>• Staff: 3 peers paired of Korean American college women and	• Medium: Mobile web technology to deliver storytelling HPV video and emails.<br>• Theory: Situation specific theoretical framework along	• Institutionalization: No Data

	HPV vaccination	• Sample size: (n=104)<br>• Recruitment: Use student leaders, pastors and social media to recruit participants<br>• Loss to follow up: Intervention group (n=9); control (n=8)	• Condition: intervention (n=54) and control group (n=50)<br>• Assessments: Two short-term assessment (post intervention and 2-month follow-up<br>• Vaccinated = 10	Physicians address common misconceptions	with storytelling and communication theory<br>• Duration: Not specific.<br>• Cost: Total cost not reported. However, each participant received $20 gift certificate and had chance to win additional $100 gift card	
**Lee et al.** (2016) USA	• Design: Pilot efficacy<br>• Outcome: Increase receipt of HPV vaccine	• Target Population: Korean American women ages 21-29<br>• Behavior: No prior receipt of HPV vaccine<br>• Sample (n = 30)<br>• Recruitment: Multi-recruitment methods including brochures, flyers, advertisement on social media.	• Vaccine uptake: 30% received first dose of vaccine.<br>• Other impact: Increase in knowledge and intent for the vaccine.<br>• Condition: Single pre/post quasi-experimental design<br>• Assessments: Baseline and post-intervention assessment (one week)<br>• Vaccinated = 9	• Setting: No one specific setting. Recruited participants from churches, clinic and other community settings<br>• Staff: Delivery staff was not described.	• Medium: Used text message, mobile phone intervention<br>• Theory: Fogg Behavioral and Trans-theoretical Models<br>• Duration: Messages delivered for 20-30 minutes each day for 7 days<br>• Cost: Not reported	• Institutionalization: No Data
**Matheson et al.** (2014) USA	• Design: Not stated<br>• Outcome: measure increase in HPV vaccine series completion rates	• Target Population: Adolescent and young adult between 11 – 22 years.<br>• Behavior: Patients and parent hospital visits and family initiated the vaccine.<br>• Sample: (n=312)<br>• Recruitment: Recruited during hospital visit	• Vaccine uptake: 14% in intervention group completed the vaccine series compared to 0% in interested group and 3% in standard care group<br>• Other impact: Increased in second doses.<br>• Condition: Not RCT: Three aims (Intervention group n=37, interested group n =43 and standard care n =232)	• Setting: Pediatric clinic<br>• Staff: Health care providers	• Medium: Third party Web-based reminder system delivered text message reminders<br>• Theory: No theory<br>• Duration: Three different text messages at different times<br>• Cost: Not reported	• Institutionalization: Conducted long term follow-up

			• Assessments: two post intervention assessment for 2 dose and 3 doses.<br>• Vaccinated =5+7=12			
**Mohanty et al.** (2018) USA	• Design: Population-based<br>• Outcome: measure increases in HPV vaccine uptake	• Target Population: Male and female adolescents ages 13-18<br>• Behavior: Under-vaccinated population<br>• Sample: (n=155,110)<br>• Recruitment: Facebook campaign was used to recruit participants	• Vaccine uptake: 152 adolescents received vaccinations<br>• Other impact: 63 participants completed 3 doses<br>• Condition: No specific comparison group.<br>• Assessments. Assessed participants activities through Facebook matrix and the hospital verifications.<br>• Vaccinated = 215	• Setting: No specific setting but participants were recruited from Philadelphia areas.<br>• Staff: Staff from the Philadelphia Department of Public Health	• Medium: Facebook and webpage were used to deliver the intervention<br>• Theory: Health Belief Model<br>• Duration: Campaign was run for two weeks period.<br>• Cost: Described the campaign cost ($3,000 per advertising campaign but did not report total cost.	• Institutionalization: No data reported.
**Ortiz et al**. (2018) USA	• Design: Not stated<br>• Outcome: measure HPV vaccine completion/uptake	• Target Population: Adolescents 13-18 years<br>• Behavior: Not yet initiated or completed HPV vaccine series<br>• Sample: (n= 108)<br>• Recruitment: Posters and fliers were used to recruited participants from community center and clinic.	• Vaccine uptake: No significant increase in HPV vaccination<br>• Other impact:<br>• Condition: Intervention group (n=82) and control group (n =26)<br>• Assessments: Baseline and three months post intervention assessments.<br>• Vaccinated. Not available	• Setting: Took place in 2 Southeastern US cities<br>• Staff: No data available	• Medium: Facebook was used to deliver the intervention.<br>• Theory: Health Belief Model<br>• Duration: During 3-month time period totaling 24 health facts<br>• Cost: Total cost not reported. However, each participant received $25 gift card.	• Institutionalization: No data reported.

**Patel et al.** (2014) USA	• Design: RCT<br>• Outcome: HPV vaccine uptake and completion	• Target Population: Women between ages 19-26<br>• Behavior: Received first dose of vaccine.<br>• Sample: (n=365)<br>• Recruitment: Participants were recruited during the hospital visit<br>• Inclusion and exclusion: Sufficiently described<br>• Participation rate: No data	• Vaccine uptake: No increase in completion rates in intervention group (17.2%) and control group (18.9%) group)<br>• Other impact: Older, college degree holder and having lifetime partner complete 3 doses<br>• Condition: cluster-randomized study (Intervention 180 vs. control 185)<br>• Assessments: Baseline assessment was based on receiving first dose of vaccination. Subsequent assessments were based on when the second or third vaccination were received.<br>• Vaccinated = 31+35=66	• Setting: 9 Planned Parenthood clinics, 1 hospital family planning clinic<br>• Staff: Recruitment was done by trained research staff at 10 family planning clinics across 7 US states but those who delivered the intervention was not described.	• Medium: women selected preferred method (text, email, phone, private Facebook message, and standard mail as reminder)<br>• Theory: No theory<br>• Duration: Each participant received 4 messages reminder (over 32 week period)<br>• Cost: No data	• Institutionalization: Followed up the participants up to 32 weeks
**Piedimonte S et al.** (2018) USA/Canada	• Design: RCT<br>• Outcome: HPV vaccine uptake; also measured 3-dose completion rate	• Target Population: University students with mean 24.79 year<br>• Behavior: Used targeted education and vaccination campaign<br>• Sample: Phase I (n=56) and phase II (n=839)<br>• Recruitment: Campaign was implemented on campuses.	• Vaccine uptake: 29 out of 56 were vaccinated in phase I and 64 of 151 were vaccinated. Additional 957 vaccinated<br>• Other impact: Increased knowledge<br>• Condition: McGill University (intervention group) and Concordia University (control)<br>• Assessments: Baseline assessment in phase I and follow-up assessments not stated but implied that they were done at the time they received vaccines<br>• Vaccinated =1,050	• Setting: Two university campuses.<br>• Staff: 2 residents, 6 medical students	• Medium: Social media advertising and mass emailing and posters on campus (did not specify which social media platforms – just social media advertising)<br>• Theory: No theory<br>• Duration: Not clearly stated<br>• Cost: Total cost was not reported but the cost of vaccination was reported.	• Institutionalization: Reported 2- and 6-months follow-up assessment.<br>• Program continued after the intervention.

		• Unintended consequences were monitored and reported.				
**Rand et al.** (2015) USA	• Design: RCT<br>• Outcomes: measure HPV vaccine dose 1: measure uptake of HPV [illegible] 2 and 3	• Target Population: Parents of unvaccinated adolescents (11-16)<br>• Behavior: No record of HPV vaccination<br>• Sample: (n=3812) [illegible] sages were used to invite participants	• Vaccine uptake: Intervention 16% vs. Control 13%<br>• Other impact: Not reported<br>• Condition: Randomized practices to intervention (n=1,893) and control group (n =1,919) [illegible] throughout the intervention period.<br>• Vaccinated 303+249=552	• Setting: 29 pediatric clinics and 10 family medicine clinics in MCO network in upstate NY<br>• Staff: Managed care [illegible] grammer delivered the intervention.	• Medium: Used text message reminder-recall system<br>• Theory: No theory<br>• Duration: Up to four text messages	• Institutionalization: Measured first, second and third doses meaning assessments were done up to 12 months or more but not [illegible].
**Rand et al.** (2017) USA	• Design: RCT<br>• Outcome: measure the time from enrollment to receipt of HPV doses 2 and 3 (for adolescents who had already started vaccine series)	• Target Population: Parents of adolescents 11-17 years<br>• Behavior: Not completed 2nd and 3rd doses of vaccine.<br>• Sample: (n = 749)<br>• Recruitment: Method not clearly stated but participants were recruited from clinics<br>• Used intent-to-treat analysis	• Vaccine uptake: 48% of phone intervention vs. 40% of phone control and 49% of text intervention vs 30% of text control had received 2 HPV vaccine doses<br>• Other impact: text message reminders and phone message reminders proved to be effective for the adolescents<br>• Condition: two parallel, two arm RCTs (phone reminder (n=178) vs standard of care (n=180), text reminder (n=191 vs standard of care n=200)<br>• Assessments: vaccine dose uptake measured during and immediately after intervention<br>• Vaccinated 85+72+94+60=311	• Setting: 3 urban primary care clinics in Rochester, NY (pediatric, medicine pediatric, family medicine).<br>• Staff: Not data	• Medium: Used phone and text message reminders<br>• Theory: No theory<br>• Duration: maximum of 3 reminders sent for each dose one week apart<br>• Cost: No data	• Institutionalization:<br>• intervention lasted 18 month period

**Title:**			Group Based	Individual	Interactive technology	Theory
**(Author, Journal, Year, Page):**			**Comments:**			
**Outcome Measures: Primary Outcomes:** knowledge and attitudes about cervical cancer prevention and on their human papillomavirus vaccination intention						

REACH	Reported (Yes/No)	Data	Comments
Described Target Population			
Demographic & behavioral information			
Method to identify target population			
Recruitment Strategies			
Inclusion criteria			
Exclusion criteria			
Target population denominator			
Sample size			
Participation rate			
Characteristics of both participation and non-participation			
Cost of recruitment			

EFFICACY/EFFECTIVENESS	Design	Conditions
**Design/Conditions**		
Efficacy, Effectiveness, Translational?		
Measure of primary outcome with or w/o comparison to a public health goal (e.g. HP 2020 goals, exercise 30 min/day; eat 5 Fruits &Veggies)		
Results (at shortest assessment)		
Intent-to-treat or present at FU (circle one)		
Imputation procedures (specify)		
Quality of life measure		
Measure unintended consequences (negative) & Results		
Measure of robustness across subgroups (e.g. moderation analyses)		
Measure of short-term attrition (%) and differential rates by patient characteristics or treatment condition		

**al.** (2016) USA	• Outcomes<br>Primary: HPV vaccine series completion	students between 18 – 26 years<br>• Behavior: had received first HPV vaccine dose<br>• Sample: (n=264)<br>• Recruitment: Participants also recruited through special health education events held by study staff.<br>• Participation rate: all participants completed baseline survey, 34% of intervention group completion f/u survey, 39% of control group completed f/u survey	HPV doses 2 and 3 not significantly different (53% for intervention grp vs 52% for control grp for HPV dose 2 and 34% for intervention grp vs 32% for control grp for dose 3)<br>• Other impact: intervention group had significantly higher mean knowledge scores<br>• Condition: Intervention (n=130) vs control (n = 134)<br>• Assessments: Baseline assessments and 7 months post intervention assessment<br>• Vaccinate 69+70=139	rural university in NC<br>• Staff: student center pharmacist approached and consented students into study, also administered HPV vaccine doses<br>• Location of intervention was well described	minders and educational messages. Control participants received standard of care (paper card with next appt date).<br>• Theory: No theory<br>• Duration: Intervention group received 7 electronic messages across 7 months<br>• Cost: Total cost not reported but each participant received $10 iTunes gift card and a chance to win an Apple iPad.	ization:<br>• baseline paper survey administered at enrollment, electronic f/u survey via Qualtrics administered 7 months after HPV dose 1 receipt
**Richman et al.** (2019) USA	• Design: RCT<br>• Outcome: HPV vaccine uptake and completion	• Target Population: Parent child dyads (child ages 9 - 17 years)<br>• Behavior: Never vaccinated<br>• Sample: (n =257)<br>• Recruitment: Participants were recruited when they were at clinic to receive first dose vaccine	• Vaccine uptake: Completion rates intervention 65% and control group 65% for HPV dose 2 and HPV dose 3 (35% vs. 30% respectively<br>• Other impact: intervention not successful at increasing HPV 3<br>• Condition: Parent child dyads randomized to intervention (n=129) vs parent child dyads randomized to control (n=128)	• Setting: 2 community clinics (1 in Pitt county, 1 in Greene county)<br>• Staff: study staff trained in basic study recruitment (Pitt county clinic)<br>• doctors/nurses who administer HPV vac-	• Medium: Used text/email appt reminders and educational messages. Control group received standard of care<br>• Theory: No theory<br>• Duration: Intervention group received 7 electronic messages<br>• Cost: Total cost not reported but each participant	• Institutionalization:<br>No data reported

			• Assessments: baseline survey administered at enrollment; electronic survey	cines trained in recruitment (Greene	received $15 Wal-Mart gift card	
Tull et al. (2019) Australia	• Design: RCT<br>• Outcome: HPV vaccine uptake of any	• Target Population: Students and parents of adolescents of year 7 students (7th grade students).<br>• Behavior: child had not yet completed HPV vaccine series<br>• Sample: (n=4386)<br>• Recruitment: Recruited parents using a list from the selected schools.<br>• Used intent-to-treat analysis	• Vaccine uptake: 85.71% of students in control, 88.35% in motivational arm, and 89.00% of students in self-regulatory arm received any dose of HPV vaccine at the third school visit<br>• Other impact: Extended follow-up effect was noticed.<br>• Intent-to-treat analysis<br>• Condition: RCT: Motivational Short Message Service (SMS) n=1442 vs. self-regulatory SMS n=1418 vs. no SMS n =1,526<br>• Assessments: study completion rates measured at end of intervention (Dec 2016) and end of extended f/u period (Jan 2017)<br>• 1308+1274+1262=3,844	• Setting: Schools<br>• Staff: No data<br>• Setting participation rate: 7 out of 12 local government immunization providers identified by research team participated<br>• 31 out of 108 schools participated	• Medium: Used text messages (groups were motivational SMS vs self-regulatory SMS vs no SMS at all)<br>• Theory: motivational strategy based on HBM<br>• Duration: 1 reminder SMS sent 2 working days before HPV vaccine visit<br>• Cost: No data	• Institutionalization: Extended f/u period for students who missed 3rd dose visit

Cost effectiveness			
**ADOPTION - Setting Level**	**Reported (Yes/No)**	**Data**	**Comments**
Description of intervention location			
Description of staff who delivered intervention			
Method to identify target delivery agent			
Level of expertise of delivery agent			
Inclusion/exclusion criteria of settings or interventionist			
Rate (#participating settings/total settings)			
Organizational spread (how far into an organization)			
Characteristics of adoption/non-adoption			
Measures of cost of adoption			
Dissemination beyond originally planned			
**IMPLEMENTATION**	**Reported (Yes/No)**	**Data**	**Comments**
Theories			

Technology/Social media			
Intervention number of contacts			
Timing of contacts			
Duration of contacts			
Extent protocol delivered as intended (%)			
Participant attendance/completion rates			
Measures of cost			
MAINTENANCE	Reported (Yes/No)	Data	Comments
Was individual behavior assessed at some duration following the completion of the intervention?<br>(give duration of follow-up)			
Attrition			
Is the program still in place?			
If no: reason for discontinuation			
If yes: was the program modified? Specify			
Was the program institutionalized?			

Measure of primary outcome (with or w/o comparison to a public health goal) at ≥ 6mo follow-up after final intervention contact			
Measure of broader outcomes or use of multiple criteria at follow- up (e.g., measure of QoL or potential negative outcome) at follow- up			
Robustness data - something about subgroup effects over the long- term			
Measure of long-term attrition (%) and differential rates by patient characteristics or treatment condition	No		
